# Risk factors for acute kidney injury in patients with complicated intra-abdominal infection

**DOI:** 10.1186/cc14364

**Published:** 2015-03-16

**Authors:** A Suarez de la Rica, E Maseda, V Anillo, C Hernandez-Gancedo, A Lopez-Tofiño, M Villagran, F Gilsanz

**Affiliations:** 1 Hospital Universitario La Paz, Madrid, Spain

## Introduction

AKI has been poorly studied in surgical septic patients. The aim of our study was to determine the factors related to AKI in surgical septic patients with complicated intra-abdominal infection (CIAI) and mortality associated with AKI in this setting.

## Methods

An observational study was performed of all adult patients with CIAI requiring surgery and ICU admission from June 2011 to June 2013. We recorded demographic data, SAPS II, SOFA score at admission, presence of septic shock, history of pre-existing comorbidities, angiotensin-converting enzyme inhibitor (ACEI), angiotensin receptor blocker (ARB), NSAIDs, statins or diuretics consumption, baseline creatinine and at admission, and standard biomarkers. Factors associated with developing AKI and renal replacement therapy (RRT) were studied using a multivariate analysis. Association between mortality and AKI and RRT need was also analyzed.

## Results

A total of 114 patients were included, with a mean SAPS II of 42.14. Sixty-seven patients (58.8%) developed AKI and 33 (28.9%) required RRT. Development of AKI (*R*^2 ^= 0.498; *P *< 0.0001; AUC = 0.926) was independently associated with SOFA (OR = 1.57; 95% CI = 1.29, 2.02) and creatinine at admission (OR for 0.1 units = 1.56; 95% CI = 1.30, 1.99). RRT need (*R*^2 ^= 0.382; *P *< 0.0001; AUC = 0.892) was independently associated with arterial hypertension (HTN) (OR = 4.90; 95% CI = 1.50, 15.97) and SOFA score (OR = 1.71). In another model with more predictive capacity (*R*^2 ^= 0.433; *P *< 0.0001; AUC = 0.918) the number of previous medications (OR = 3.73; 95% CI = 1.92, 8.38) and SOFA score (OR = 1.86; 95% CI = 1.47, 2.54) were related to RRT need. Both AKI and RRT need were related to ICU (RR = 8.41, 95% CI = 1.14, 62.5; and RR = 8, 95% CI = 2.40, 27.85 respectively) and 28-day mortality (RR = 2.8, 95% CI = 1.00, 7.86; and RR = 4.65, 95% CI =1.99, 10.40 respectively).

## Conclusion

Severe AKI with RRT need is highly associated with previous HTN. The number of previous medications is related to severe AKI too. HTN has been described as a risk factor for developing AKI in critically ill patients [1]. ACEI and ARB use has been associated with AKI development in septic patients [2]. To our knowledge, this is the first study that investigates risk factors associated with AKI in surgical septic patients with CIAI.
